# IL-37 inhibits the production of inflammatory cytokines in peripheral blood mononuclear cells of patients with systemic lupus erythematosus: its correlation with disease activity

**DOI:** 10.1186/1479-5876-12-69

**Published:** 2014-03-16

**Authors:** Liang Ye, Ling Ji, Zhongyang Wen, Yanfei Zhou, Dongsheng Hu, Yanqun Li, Ting Yu, Bingni Chen, Jinshun Zhang, Liping Ding, Jing Du, Zhong Huang

**Affiliations:** 1 Biological therapy institute, Shenzhen University School of Medicine, 518060 Shenzhen, China; 2 Department of Pathogen Biology and Immunology, Shenzhen University School of Medicine, 518060 Shenzhen, China; 3 Shenzhen City Shenzhen University Immunodiagnostic Technology Platforms, 518060 Shenzhen, China; 4 Department of laboratory medicine, Peking University Shenzhen Hospital, 518036 Shenzhen, China; 5 Department of Preventive Medicine, Shenzhen University School of Medicine, 518060 Shenzhen, China

**Keywords:** Interleukin-37, Systemic lupus erythematosus, Autoimmunity, Cytokines, Peripheral blood mononuclear cell

## Abstract

**Background:**

Interleukin-37 (IL-37), a new member of IL-1 family cytokine, is recently identified as a natural inhibitor of innate immunity. This study aimed to measure the peripheral blood mononuclear cells (PBMCs) and serum levels of IL-37 in patients with systemic lupus erythematosus (SLE) and to investigate its role in SLE, including its correlation with disease activity, organ disorder and the regulation of inflammatory cytokines.

**Methods:**

The expressions of IL-37 mRNAs in PBMCs and serum IL-37 levels in 66 SLE patients were measured by real-time polymerase chain reaction (RT-PCR) and enzyme-linked immunosorbent assay (ELISA). SLE patients PBMCs were stimulated with recombinant IL-37, levels of cytokines TNF-α, IL-1β, IL-6 and IL-10 were detected by RT-PCR and ELISA.

**Results:**

IL-37 mRNAs and serum protein levels were higher in patients with SLE compared with healthy controls. Patients with active disease showed higher IL-37 mRNAs and serum protein levels compared with those with inactive disease as well as healthy controls. Serum IL-37 levels correlated with SLEDAI and inversely with C3 and C4. Serum IL-37 levels were higher in SLE patients with renal involvement compared with those without renal disease. *In vitro*, IL-37 inhibited the production of TNF-α, IL-1β and IL-6 in PBMCs of patients with SLE, whereas the production of IL-10 was unaffected.

**Conclusions:**

IL-37 associated with SLE disease activity, especially related with SLE renal disease activity. IL-37 is an important cytokine in the control of SLE pathogenesis by suppressing the production of inflammatory cytokines. Thus, IL-37 may provide a novel research target for the pathogenesis and therapy of SLE.

## Background

The interleukin (IL)-1 family of cytokines possesses a variety of immunoregulatory properties in response to inflammation and autoimmune diseases [1]. As the members of the IL-1 family, seven cytokines (IL-1α, IL-1β, IL-18, IL-33, IL-36α, IL-36β and IL-36γ) act as agonists, and two are classified as naturally occurring receptor antagonists (IL-1Ra and IL-36Ra) [2,3]. IL-37, the most recently identified IL-1 member, originally defined as IL-1 family member 7 (IL-1F7), is a fundamental inhibitor of innate immunity [4].

Recent studies demonstrated that anti-inflammatory cytokines TGF-β, IL-10, several toll-like receptor (TLR) ligands and pro-inflammatory cytokines such as IL-1β, TNF-α, IFN-γ and IL-18 induce IL-37 production in peripheral blood mononuclear cells (PBMCs) [4,5]. However, over-expressed human IL-37 suppressed the TLR-induced pro-inflammatory cytokines in a mouse macrophage RAW cell line, in human monocyte cell line THP-1 and in alveolar epithelial A549 cells [1,4]. *In vivo,* mice transgenic for IL-37 (IL-37tg) exhibited markedly reduced manifestations of DSS colitis, ischemia–reperfusion injury and obesity-induced inflammation [6-8]. Compared to health subjects, IL-37 was constitutively expressed in tissues from patients with rheumatoid arthritis [4]. Similar studies have found that IL-37 was not detected in the normal colonic mucosa, but in the inflamed mucosa of IBD patients [9]. These findings tend to imply that IL-37 mediates a negative feedback mechanism to suppress excessive inflammation.

Systemic lupus erythematosus (SLE) is an autoimmune and inflammatory disease characterized by the activation of T and polyclonal B lymphocytes. The activation of B cells produces numerous auto-antibodies and form immune complexes with variety antigens, which result in tissue and organ damage [10]. Cytokines collectively play key roles in the regulation of systemic inflammation, local tissue damage and immunoreactions [11]. Abnormal release various cytokines have been identified in SLE patients and animal models both *in vitro* and *in vivo*[12]. Abundant studies have demonstrated that pro- and anti-inflammatory cytokines, including TNF-α, IL-1β, IL-6 and IL-10, play crucial pathogenic roles in the disease of SLE [12,13]. Although the serum IL-37 levels have been found to be elevated in active patients with SLE [14], its causal relationship with disease activity and their clinical association and disease manifestations in SLE is still unclear, especially the regulations of cytokines expression by IL-37 in SLE PBMCs remain to be studied.

In present study, we compared expressions of IL-37 mRNAs in PBMCs and serum IL-37 protein levels in SLE patients with healthy controls. In addition, we determined the correlation of serum IL-37 levels with disease activity and clinical manifestations in SLE, investigated the effect of IL-37 on the expressions of cytokine TNF-α, IL-1β, IL-6 and IL-10.

## Materials and methods

### Patients and controls

The study was approved by the Review Board for Peking University Shenzhen Hospital in Shenzhen, People’s Republic of China. Sixty-six patients who satisfied the revised classification criteria of the ACR for SLE [15] were recruited from the Rheumatology Department, Peking University Shenzhen Hospital. Forty-one age- and sex-matched volunteers were recruited from Peking University Shenzhen Hospital as healthy controls. Informed consent was obtained from recruited subjects. Individuals with other rheumatic diseases, infections or malignant tumors were excluded from the study.

SLE patients’ laboratory tests containing anti-double stranded (ds) DNA antibody, anti-nucleosome antibody (AnuA), anti-smith antibody (Smith), Anti-Ribosomal RNP Antibody (rRNP), erythrocyte sedimentation rate (ESR), complement 3 (C3), complement 4 (C4), IgG, IgM and IgA were performed. Clinical data from each patients were recorded. Lupus disease activities were assessed by using the Systemic Lupus Erythematosus Disease Activity Index (SLEDAI) score [16]. Active lupus disease was defined as SLEDAI score ≥ 6 [17,18]. Demographic characteristics of the SLE patients and healthy controls are listed in Table 1.

**Table 1:** Demographic and clinical characteristics at the time of study of SLE and healthy controls

**Characteristics**	**SLE patients (n = 66)**	**Healthy controls (n = 41)**
Age (years)	30.5 ± 8.02 (18-54)	31.8 ± 9.57 (19-58)
Sex (female/male)	59/7	37/4
Disease duration (years)	2.0 ± 1.12	-
Arthritis n (%)	3 (4.5)	-
Renal diseases n (%)	40 (60.6)	-
Fever n (%)	13 (19.7)	-
Neurological disorder n (%)	1 (1.5)	-
Leukopenia n (%)	11 (16.7)	-
Thrombocytopenia n (%)	8 (12.1)	-
Anti-ds-DNA antibody n (%)	39 (59.1)	-
Anti-Smith antibody n (%)	30 (45.5)	-
Anti-AnuA antibody n (%)	20 (30.3)	-
Anti-rRNP antibody n (%)	11 (16.7)	-
ESR	29.2 ± 27.80	-
Low C3 n (%)	33 (50.0)	-
Low C4 n (%)	29 (43.9)	-
IgG (g/L)	16.2 ± 5.63	-
IgM (g/L)	1.21 ± 0.64	-
IgA (g/L)	2.33 ± 1.09	-
SLEDAI	(2-14) 6.34 ± 3.26	-

Except where otherwise indicated, values are expressed as mean ± standard deviation. There were no significant differences between patients with SLE and healthy donors in terms of age and sex. Anti-ds-DNA antibody, anti-double stranded DNA antibody; Anti-Smith antibody, anti-smith-antibody; Anti-AnuA antibody, anti-nucleosome antibody; Anti-rRNP antibody, Anti-Ribosomal RNP Antibody; ESR, erythrocyte sedimentation rate; C3, complement 3; C4, complement 4; IgG, Immunoglobulin G; IgM, Immunoglobulin M; IgA, Immunoglobulin A; SLE, systemic lupus erythematosus; SLEDAI, SLE disease activity index.

### Blood samples

Fasting venous bloods (4 ml) were collected and processed within three hours. PBMCs were isolated from patients and healthy controls by a Ficoll-Paque Plus (TBDscience, China) density gradient centrifugation for cell culture or stored at -80°C until RNA extraction. Serum samples were stored at -80°C until cytokine were determined.

### Recombinant human IL-37 protein

#### Cloning

The gene for interleukin 37 gene (homo species) was amplified from cDNA of peripheral blood mononuclear cell using the primer pair 5′- CGGGATCCATGGTTCACACAAGTCCA-3′ and 5′- CCCAAGCTTCTAATCGCTGACCTCACT-3′. The PCR fragments were double digested with restriction endonucleases (Takara, China) and ligated into the prokaryotic expression vectors.

#### Expression

The fusion proteins were expressed in a stable prokaryotic expression system. Briefly, ligation mixtures (as described above) were transformed into E. coli Trans (T1) competent cells. The positive clones were identified by colony PCR. The plasmids of positive clones were then sequenced by Sanger method. The plasmid that was 100% identify with the published sequence (GenBank:AF167368) was transformed into expression host E. coli competent cells. The induced and uninduced cultures were analyzed by SDS-PAGE to identify the expression of recombinant protein.

#### Purification and characterization of IL-37

The harvested cells were resuspended in NaCl- Tris-HCl buffer, sonicated in an ice bath, 12000 rpm centrifuged 30 min, then the supernatant were collected. The supernatant were added to His Trap HP, 1 ml column (GE) that had been equilibrated with NaCl-Tris-HCl buffer. Different concentrations of imidazole buffer were used to elute the recombinant protein. Collected target protein peaks were examined by SDS-PAGE electrophoresis and immunoblot analysis using anti-human IL-37 antibodies (Abcam, UK). The eluted recombinant proteins were dialyzed in PBS at 4°C for overnight. The concentrations were detected by Brandford methods, and the recombinant protein was stored at -20°C.

### Cell culture condition

Culture medium, consisted of RPMI 1640 (Hyclone, Thermo, USA) supplemented with 10% Fetal Calf Serum (Sijiqing, Zhejiang, China), 100 IU/ml penicillin and 100 μg/ml streptomycin (Beyotime, China), respectively. Whole PBMCs were cultured in 24 well, flat-bottomed plates (5 × 10^5^ cells/ml/well) for 24 h, PBMCs were stimulated with or without human recombinant IL-37 at 100 ng/ml for 6 h, total RNAs were extracted, and cytokine transcriptions were analyzed by RT-PCR. PBMCs were stimulated with or without human recombinant IL-37 at 100 ng/ml for 24 h, and then incubated further with LPS (1 μg/ml) for 6 h, culture supernatants were harvested and froze at -80°C for later cytokine analysis by ELISA.

### RNA extraction and real-time polymerase chain reaction (RT-PCR)

Total RNA was extracted from PBMCs with Trizol (Invitrogen, Carlsbad, CA, USA) according to the manufacturer’s instructions. Then the quantity and purity of RNA was determined by absorbance on a FilterMax F5 Multi-Mode Microplate Reader (Sunnyvale, California, USA) at 260 nm and 280 nm. Samples with ratios from 1.8 to 2.0 were accepted for next reverse transcription reaction. cDNAs were prepared by using the iScript™ cDNA Synthesis kit (Bio-Rad, USA). PCR primers (Generay, Shanghai, China) used for RT-PCR were as follows: for IL-37, sense: 5′-AGTGCTGCTTAGAAGACCCGG-3′ and 5′-AGAGTCCAGGACCAGTACTTTGTGA-3′(reverse); TNF-α, sense: 5′-ACCTCTCTCTAATCAGCCCTCT-3′ and anti-sense: 5′-GGGTTTGCTACAACATGGGCTA; IL-1β, sense: 5′- CCACAGACCTTCCAGGAGAAT-3′ and anti-sense: 5′-GTGCACATAAGCCTCGTTATCC-3′; IL-6, sense: 5′-AGCCACTCACCTCTTCAGAAC-3′ and anti-sense: 5′-ACATGTCTCCTTTCTCAGGGC-3′; IL-10, sense: 5′-CTGCCTAACATGCTTCGAGATC-3′ and anti-sense: 5′-TCCTCCAGCAAGGACTCCTTT-3′; β-actin, 5′-CCTGACTGACTACCTCATGAAG-3′ and anti-sense: 5′-GACGTAGCACAGCTTCTCCTTA-3′. RT-PCR amplification reactions were prepared with the SYBR Green PCR kit (Bio-rad, USA) and performed using the 7500 fast Real-Time PCR system (Applied Biosystems, USA). PCR products were verified by melting curve analysis. Relative mRNA levels of target genes were calculated by the 2^-ΔΔct^ method.

### Enzyme-linked immunosorbent assay (ELISA)

Serum IL-37 levels and cell culture supernatant IL-37, TNF-α, IL-1β, IL-6 and IL-10 levels were determined by ELISA following the manufacturer’s instructions. IL-37 was quantified using ELISA reagent kits purchased from AdipoGen (San Diego, CA, USA). Detection of the cytokines TNF-α, IL-1β, IL-6 and IL-10 were accomplished using the eBioscience kit (San Diego, CA, USA).

### Statistical analysis

Data were expressed as mean (±SEM) or median (range) and analysed by Graphpad Prism V.5.00 software (GraphPad Software, San Diego CA, USA). Comparisons between groups were made using nonparametric Mann-Whitney U-test. Spearman correlation test was used to assess the association between serum IL-37 levels and different variables. P values under 0.05 were considered statistically significant.

## Results

### IL-37 mRNAs and serum protein levels were higher in patients with SLE compared with healthy controls

**Figure 1:**
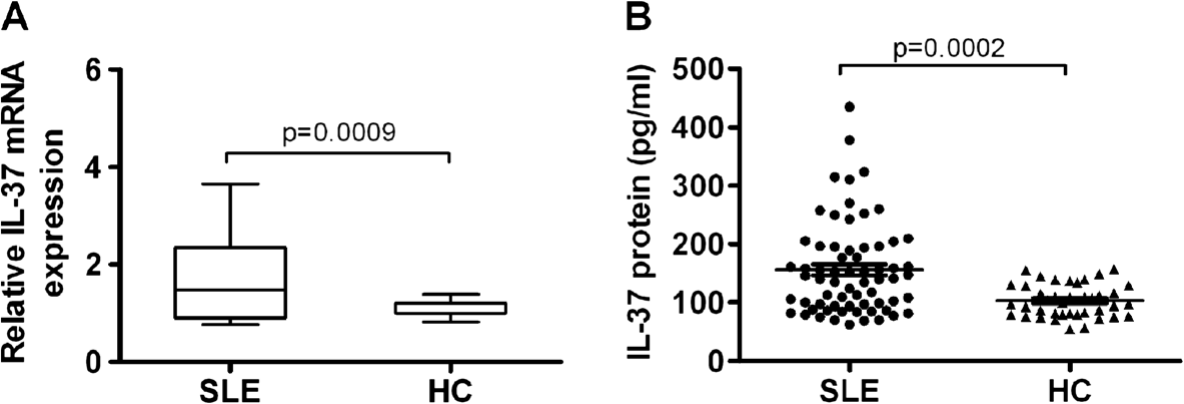
**Comparison of IL-37 mRNAs and protein levels between SLE and HC. (A)** Expressions of IL-37 mRNAs in PBMCs from SLE patients (n = 66) and healthy controls (HC, n = 41) were determined by RT-PCR. Results are depicted as box plots, with median (horizontal line within each box) and 10^th^, 25^th^, 75^th^, and 90^th^ percentiles (bottom bar, bottom of box, top of box, and top bar, respectively). **(B)** Serum IL-37 protein levels in SLE patients (n = 66) and healthy controls (HC, n = 41) were determined by ELISA. Each symbol represents an individual SLE patients and healthy controls. Horizontal lines indicate median values. Mann-Whitney U-test and associated *P* values are indicated.

The expressions of IL-37 mRNAs in PBMCs and Serum IL-37 protein levels from 66 SLE patients and 41 age- and sex-matched healthy controls (HC) were measured by RT-PCR and ELISA, respectively. SLE patients and healthy controls did not reveal significant differences in terms of mean age or sex distribution (Table 1). As shown in Figure 1A, SLE patients had significantly higher IL-37 mRNAs levels than healthy controls (P = 0.0009). Figure 1B also displayed significant elevation of serum IL-37 protein levels in patients with SLE compared with healthy controls (P = 0.0002), indicating that IL-37 probably participated in the mediation of pathogenesis of SLE.

### IL-37 mRNAs and serum protein levels were higher in SLE patients with active disease compared with those with inactive disease

**Figure 2:**
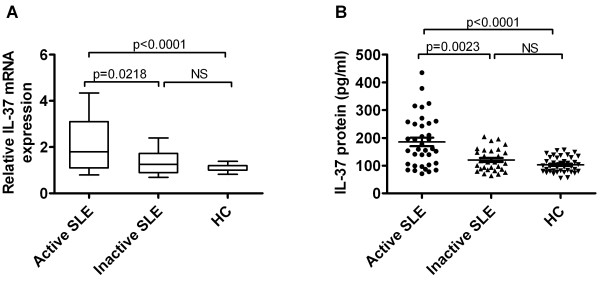
**Comparison of IL-37 mRNAs and protein levels among SLE patients with active disease and inactive disease as well as HC. (A)** Levels of IL-37 mRNAs in PBMCs from SLE patients, distributed according to disease activity (i.e. active (n = 36) versus inactive (n = 30)), were compared by RT-PCR with those from healthy controls (HC, n = 41). Results are depicted as box plots, with median values, 25^th^ and 75^th^ quartile and the range of values. **(B)** Serum IL-37 protein levels in SLE patients, distributed according to disease activity (i.e. active (n = 36) versus inactive (n = 30)), were compared by ELISA with those from healthy controls (HC, n = 41). Each symbol represents an individual patient. Horizontal lines indicate median values. Mann-Whitney U-test and associated *P* values are indicated. NS, no significant.

We next investigated whether IL-37 was related to disease activity in SLE patients. We divided SLE patients into active groups (SLEDAI score ≥ 6) and inactive groups (SLEDAI score < 6) according to SLEDAI. As seen in Figure 2A and B, significant differences were viewed in IL-37 mRNAs and protein levels between patients with active and those with inactive diseases (P = 0.0218, P = 0.0023, respectively). In the meantime, patients with active disease displayed higher IL-37 mRNAs and serum IL-37 protein levels than healthy controls (P < 0.0001, P < 0.0001, respectively). However, we did not observe the differences of IL-37 mRNAs and protein levels between patients with inactive disease and healthy controls (Figure 2). Thus, we speculated that IL-37 probably was associated with disease activity of SLE.

### Correlation between IL-37 levels and SLEDAI as well as laboratory values

**Figure 3:**
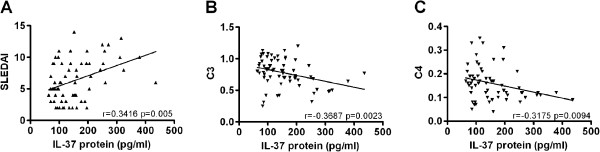
**Correlation of serum IL-37 levels with SLEDAI as well as laboratory values.** Each symbol represents an individual SLE patient. **(A)** Serum IL-37 levels were positively correlated with SLEDAI. **(B)** Negative relationship was observed between serum IL-37 levels and C3. **(C)** Negative relationship was observed between serum IL-37 levels and C4. The correlations were evaluated with Spearman’s non-parametric test.

To further survey the relationship between serum IL-37 protein levels and disease activity, we next detected correlations between IL-37 and SLEDAI as well as laboratory values such as anti-dsDNA, smith, AnuA, rRNP antibody, IgG, IgM, IgA, ESR, C3 and C4. A significantly positive correlation was observed between serum IL-37 levels and SLEDAI (r = 0.3416, P = 0.005, Figure 3A). There was a negative correlation between serum IL-37 and C3 levels (r = -0.3687, P = 0.0023, Figure 3B), as well as C4 levels (r = -0.3175, P = 0.0094, Figure 3C). No significant correlations were found between serum IL-37 levels and anti-dsDNA, smith, AnuA, rRNP antibody, IgG, IgM, IgA and ESR, respectively (data not shown).

### Association of serum IL-37 protein levels with clinical features in SLE

**Figure 4:**
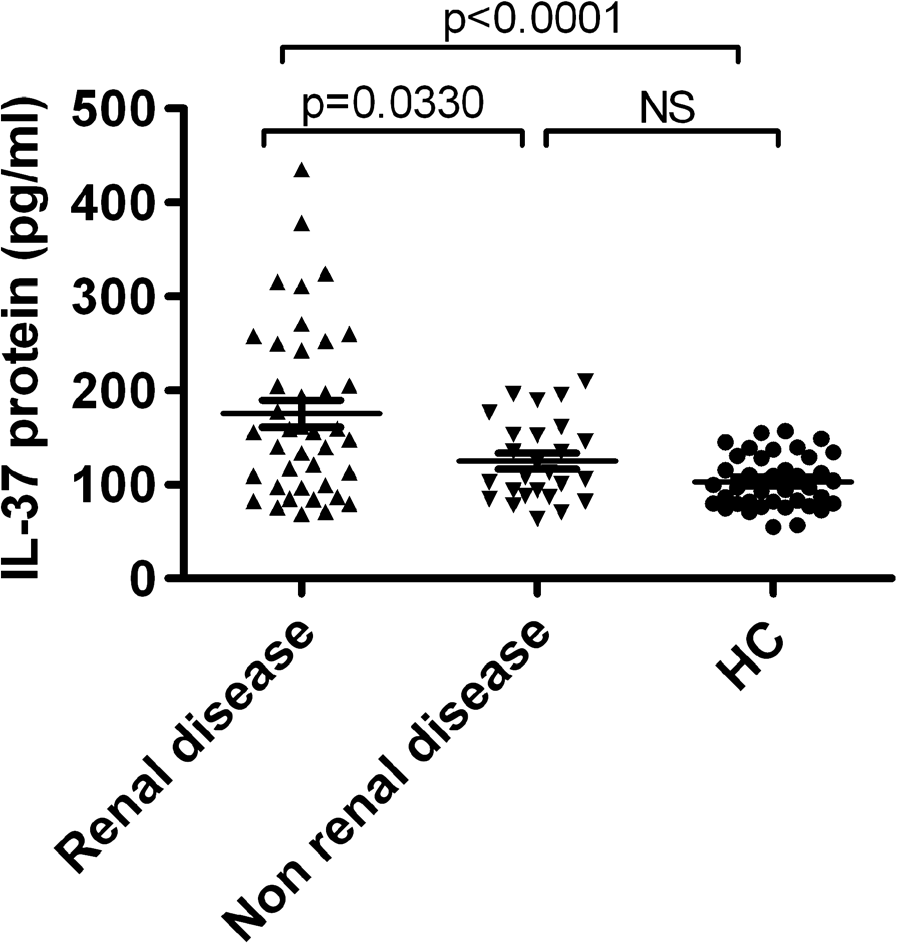
**Elevated serum IL-37 levels in SLE patients with renal disease.** Serum IL-37 levels exhibited a significant elevation in patients with renal involvement (n = 40) relative to patients without renal disease (n = 26) as well as healthy control (HC, n = 41). Each symbol represents an individual patient or healthy control. Horizontal lines indicate median values. Mann-Whitney U-test and associated *P* values are indicated. NS, no significant.

To assess associations between serum IL-37 protein levels and clinical manifestations, serum IL-37 protein levels were compared among patients with and those without certain clinical features as well as healthy controls. We identified that no significant differences in serum IL-37 protein levels between patients in the presence of malar rash, serositis, arthritis, fever, neurological disorder, anemia, leucopenia and thrombocytopenia, and patients in the absence of the above-mentioned clinical manifestations (data not shown). Nevertheless, we discerned that serum IL-37 levels were significantly higher in patients with renal disease compared with those without these manifestations (P = 0.033, Figure 4), furthermore the patients without renal disease did not show significant higher serum IL-37 level than healthy controls (Figure 4), illustrating that IL-37 probably related with renal disorder in SLE.

### IL-37 inhibits the productions of inflammatory cytokines in PBMCs of SLE patients

**Figure 5:**
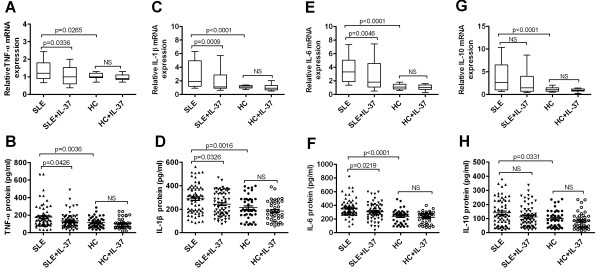
**IL-37 inhibits the expression of inflammatory cytokines in PBMCs of patients with SLE.** PBMCs from SLE patients (n = 66) and healthy controls (n = 41) were stimulated with IL-37 (100 ng/ml) for 6 hour, the total RNAs were extracted and analyzed for TNF-α **(A)**, IL-1β **(C)** and IL-6 **(E)** IL-10 **(G)** mRNAs by RT-PCR. Box plots show the median values, 25^th^ and 75^th^ quartile and the range of values. The cells were stimulated with IL-37 (100 ng/ml) for 24 hour, and then incubated further with LPS (1 μg/ml) for 6 h, supernatants were examined for TNF-α **(B)**, IL-1β **(D)**, IL-6 **(F)** and IL-10 **(H)** levels using enzyme-linked immunosorbent assay (ELISA). Each symbol represents an individual patient or healthy control. Horizontal lines indicate median values. Actual *P* values are shown in the graph. NS, no significant.

IL-37 has been reported as an inhibitor of innate immunity [4]. To assess whether IL-37 had a similar capacity to regulate the expressions of inflammatory cytokines involved in the pathogenesis of SLE. We have expressed and purified recombinant human IL-37 protein (Additional file 1). Initially, we evaluated the effects of IL-37 on LPS-induced cytokines expression in PBMCs. The cells were pre-incubated with or without different concentrations of IL-37 (0 ng/ml, 1 ng/ml, 10 ng/ml, 100 ng/ml and 200 ng/ml) for 6 h, and then incubated further with LPS (1 μg/ml) for 6 h. IL-37 significantly reduced the LPS-induced TNF-α, IL-6 and IL-1β mRNA expressions at 100 ng/ml and 200 ng/ml (Additional file 2A-C), but the expression of IL-10 mRNA was not clearly to be inhibited (Additional file 2D).To further address the effect of IL-37 on cytokines in PBMCs from SLE patients, IL-37 (100 ng/ml) was added to the cultures of PBMCs from SLE and healthy controls separately. Our experiments showed that the expressions of pro-inflammatory cytokines TNF-α, IL-6 and IL-1β mRNAs in PBMCs of SLE patients were significantly suppressed by IL-37 (Figure 5A,C,E). Meanwhile, IL-37 also markedly reduced the secretions of TNF-α, IL-6 and IL-1β in PBMCs of SLE patients (Figure 5B,D,F). By contrast, no change of IL-10 mRNAs and protein levels were detected (Figure 5G and H). Interestingly, these cytokines expressions in PBMCs of healthy controls were unaffected by treatment with IL-37 (data not shown).

## Discussion

Although IL-37 mRNA expression and serum protein level has been demonstrated to be higher in active SLE patients [14], but information with regard to its clinical association and disease manifestation is lacking. Our results provide a detailed analysis of IL-37 expression in SLE patients (SLEDAI ≥ 6, active SLE and SLEDAI < 6, inactive SLE) and healthy controls. We demonstrated that IL-37 mRNAs expressions and serum protein levels were higher in 66 patients with SLE than in 41 healthy controls (Figure 1). Further investigation revealed that IL-37 mRNAs and protein levels were significantly higher in 36 patients with active diseases than in 30 patients with inactive diseases and in 41 healthy controls. However, there is no difference between inactive diseases and healthy controls (Figure 2). In order to further proved the relationship between IL-37 expression and SLE laboratory values, we made efforts to analyze the correlation between serum IL-37 protein levels and several laboratory values. Our novel data suggested that serum IL-37 levels were negatively correlated with C3 and C4 (Figure 3), but it lacked association with other laboratory values (anti-dsDNA, smith, AnuA, rRNP antibody, IgG, IgM, IgA and ESR) (data not shown). Thus, our findings implied that the expression of IL-37 is correlated with activity of SLE, and IL-37 probably involved in the mediation of the disease activity. In patients with SLE, skin involvement, arthritis and renal disorder are very common manifestations. We showed that no significant differences in serum IL-37 protein levels between the patients who had malar rash, serositis, arthritis, fever, neurological disorder, anemia, leucopenia and thrombocytopenia and the patients who absented the above-mentioned clinical manifestations (data not shown). Surprisingly, serum IL-37 levels were significantly higher in patients with renal disease compared with patients without renal involvement together with healthy controls (Figure 4). In summary, our experiments demonstrated that the expression levels of IL-37 correlated with the disease activity, laboratory values and clinical manifestations of SLE.

Imbalances between pro- and anti-inflammatory cytokines are hallmarks of the pathogenesis of SLE [19,20]. It has been demonstrated that pro-inflammatory cytokines TNF-α, IL-1β, IL-6 and cytokine IL-10 levels are significantly elevated in the serum of SLE patients and correlate with disease activity [13,21,22]. In particular, the expressions of TNF-α, IL-6 and IL-10 are markedly elevated in SLE patients with lupus nephritis [20-22]. Published data have shown that blocking these cytokines significantly improved the development of SLE [23-26]. Uppal SS *et al* showed that nine SLE patients treated with anti-TNF-α monoclonal antibody, five patients showed improvement in disease activity (SLEDAI) [24]. Two very small uncontrolled studies of three and four SLE patients suggested that blocking IL-1 signaling might have beneficial effects on lupus-related manifestations [24]. In addition, SLE patients with tocilizumab (anti-IL-6 receptor antibody) treatment resulted in significant clinical improvement, especially in SLE patients with arthritis [25]. Another study found that administration of anti-IL-10 monoclonal antibody (21 consecutive days) to six patients with moderate lupus (an open study) resulted in healing of arthritis and cutaneous lesions [26].

In our present study, the expressions of TNF-α, IL-1β, IL-6 and IL-10 were significantly increased in PBMCs of SLE patients than in PBMCs from healthy controls. Interestingly, our result also showed that the expression of IL-37 was correlated with the activity of disease in SLE. What are the functions of a fundamental innate immunity inhibitor IL-37 [4] in SLE? To answer the question, recombinant purified IL-37 was used to stimulate PBMCs from SLE patients and health controls. Our study is the first to reveal that IL-37 could effectively decrease the productions of pro-inflammatory cytokines TNF-α, IL-6 and IL-1β (Figure 5A-F), whereas the productions of cytokine IL-10 were unaffected in PBMCs of SLE patients (Figure 5G-H). Researcher showed that IL-37 can be up-regulated by cytokines IL-1β, TNF-α and IL-10 [4,5], indicating these cytokines act as positive feedback loops for up-regulation of IL-37 production. Therefore, it is reasonable to explain the correlation between higher activity of disease and the higher expression levels of IL-37 in SLE. Recently, IL-37 has been shown to inhibit the production of pro-inflammatory cytokines TNF-α, IL-1β and IL-6 in PBMCs *in vitro*[4]. Together with this result, the down regulation of pro-inflammatory cytokines in our experiments imply that IL-37 may play an important role in the inhibition of inflammatory response in SLE, the up regulation of IL-37 by SLE immune reaction may be the result of inflammatory diseases self-limiting. It has been shown that the expression of IL-10 in PBMCs was unaffected by treatment with siIL-37 [4], which was consistent with our finding. These data demonstrated that IL-37 acts as an inhibitor of inflammatory responses in SLE disease, but this function does not extend to cytokines IL-10.

Interestingly, the pro-inflammatory cytokine productions in PBMCs of healthy controls and inactive disease with SLE patients were unaffected by treatment with IL-37 (Figure 5), suggesting that anti-inflammatory actions of IL-37 are only in the inflammatory phase and active disease conditions of SLE. Nold *et al.* showed that IL-37 suppresses the phosphorylation of p38 MAPK [4], which are known to be involved in the pathogenesis of SLE and contributes to several pro-inflammatory signaling cascades [27]. It has been demonstrated that inhibition of p38 MAPK activation reduced serum TNF-a, IL-1β and IL-6 levels and attenuated SLE renal injury [28,29]. Thus, it is plausible that down-regulation of several kinase activity by IL-37, such as p38 MAPK, might result in reduced expression of pro-inflammatory cytokines in PBMC during inflammatory responses, which could lead to suppression of excessive immune response, and protection of tissue damage in SLE.

## Conclusions

In summary, our study showed that the expressions of IL-37 correlated with the disease activity of SLE. Meanwhile, it was likely to mediate a negative feedback mechanism to suppress excessive inflammation in SLE, especially in renal disease of SLE. Furthermore, we demonstrated that the expressions of pro-inflammatory cytokines TNF-α, IL-6 and IL-1β were suppressed by IL-37 in active SLE patients. These observations implicate that IL-37 probably played an important role in the inhibition of pathogenesis of SLE. Future efforts need to define the regulatory mechanisms of IL-37 in the mediation of immune reaction of SLE.

## Abbreviations

IL-37: Interleukin-37; SLE: Systemic lupus erythematosus; SLEDAI: Systemic lupus erythematosus disease activity index; PBMC: Peripheral blood mononuclear cell; PCR: Polymerase chain reaction; RT-PCR: Real-time polymerase chain reaction; ELISA: Enzyme-linked immunosorbent assay; TNF: Tumor necrosis factor; Anti-ds-DNA antibody: Anti-double stranded DNA antibody; Anti-anuA antibody: Anti-nucleosome antibody; Anti-smith antibody: Anti-smith-antibody; Anti-rRNP antibody: Anti-ribosome ribonucleoprotein antibody; ESR: Erythrocyte sedimentation rate; C3: Complement 3; C4: Complement 4; IgG: Immunoglobulin G; IgM: Immunoglobulin M; IgA: Immunoglobulin A.

## Competing interests

The authors declare that they have no competing interests.

## Authors’ contributions

LY contributed to the experimental design, data acquisition, data analysis and the manuscript draft and edition. YW, YL, TY, BC, JZ and LD performed the data acquisition and interpretation of cell culture, RT-PCR and ELISA. LY and SH participated in data analysis. FZ provided recombinant human IL-37 fusion protein. LJ and JD supplied samples, clinical and laboratory data. ZH designed experiments, analyzed the data, read and revised the draft paper. All authors read and gave final approval of the version to be published.

## Supplementary Material

Additional file 1 **Recombinant human IL-37 fusion protein.** (A) Cloning of human IL-37 gene. Human IL-37 gene was amplified using Taq polymerase and the PCR product was found about 522 bp. Molecular weight makers and sizes were shown on the left. Lane 1-5: Positive clones of human IL-37 gene; Lane 6: negative control. (B) Expressions of human IL-37 in E. coli Transetta (DE3) cells. The expression of human IL-37 was induced with IPTG. Induced and uninduced cultures were compared by SDS-PAGE. Molecular weight makers and sizes are shown on the left. Lane 1: uninduced cells; Lane 2 and 3: induced cells respectively. (C) Gel electrophoresis of purified human IL-37. Molecular weight makers and sizes are shown on the left. (D) Western blot analysis using monoclonal antibodies (mAb) against the human IL-37. Click here for file

Additional file 2 **Dose-dependent effects of IL-37 on inflammatory cytokines mRNA expression in PBMCs of healthy donors.** PBMCs of healthy donors were stimulated for 6 h with different concentrations of IL-37, and then incubated further with or without LPS (1 μg/ml) for 6 h. The TNF-α (A), IL-6 (B), IL-1β (C) and IL-10 (D) mRNAs expression was analyzed by real-time polymerase chain reaction (PCR). Values are the mean ± SEM (n = 3). *P < 0.05; **P < 0.01. Click here for file
