# Supplementary figures and images for: IL-37 inhibits the production of inflammatory cytokines in peripheral blood mononuclear cells of patients with systemic lupus erythematosus: its correlation with disease activity
Source: J Transl Med. 2014 Mar 16;12:69. doi: 10.1186/1479-5876-12-69 (PMC4003851; doi:10.1186/1479-5876-12-69)

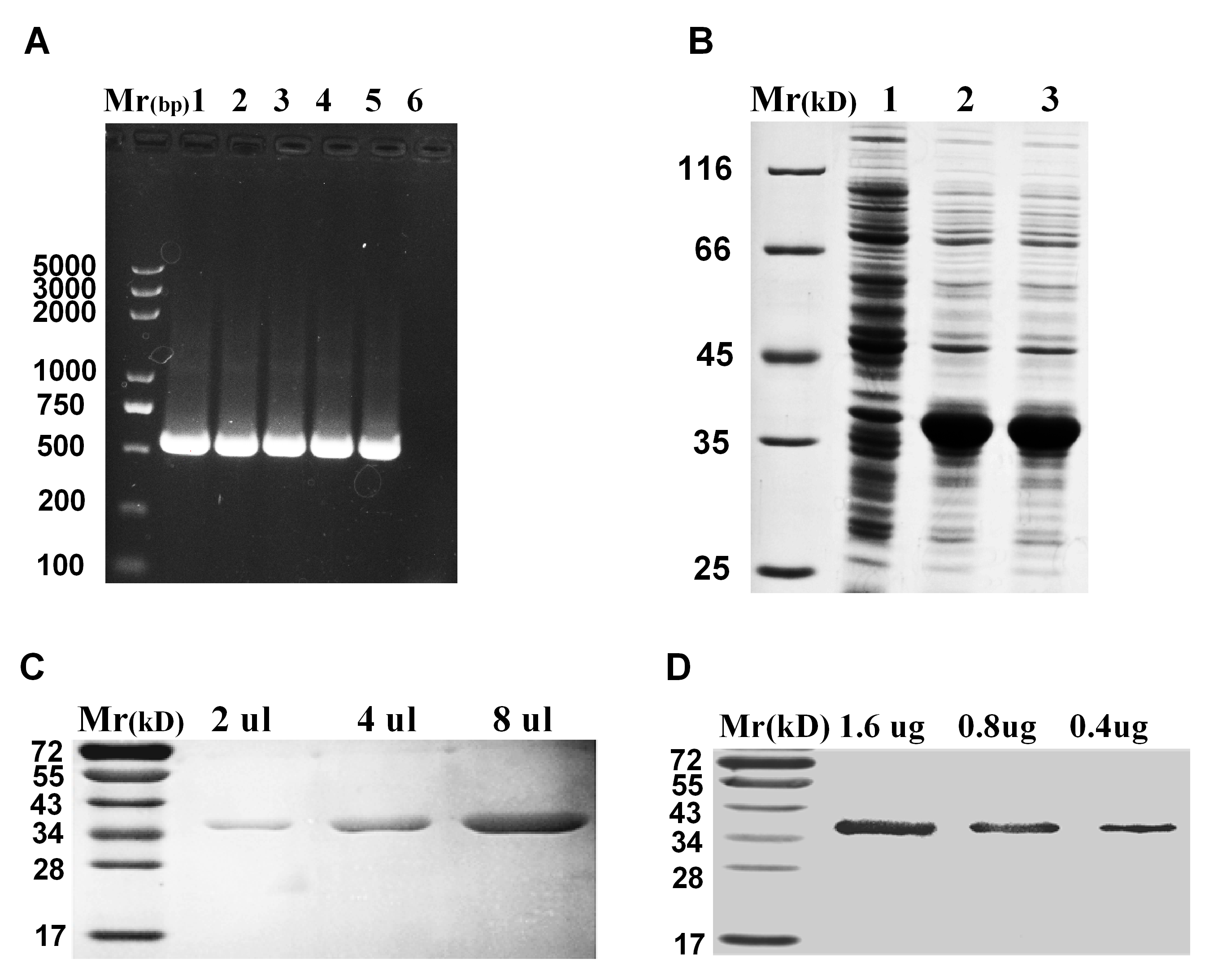

Supplement: Additional file 1 — Recombinant human IL-37 fusion protein. (A) Cloning of human IL-37 gene. Human IL-37 gene was amplified using Taq polymerase and the PCR product was found about 522 bp. Molecular weight makers and sizes were shown on the left. Lane 1-5: Positive clones of human IL-37 gene; Lane 6: negative control. (B) Expressions of human IL-37 in E. coli Transetta (DE3) cells. The expression of human IL-37 was induced with IPTG. Induced and uninduced cultures were compared by SDS-PAGE. Molecular weight makers and sizes are shown on the left. Lane 1: uninduced cells; Lane 2 and 3: induced cells respectively. (C) Gel electrophoresis of purified human IL-37. Molecular weight makers and sizes are shown on the left. (D) Western blot analysis using monoclonal antibodies (mAb) against the human IL-37. [file 1479-5876-12-69-S1.tiff]

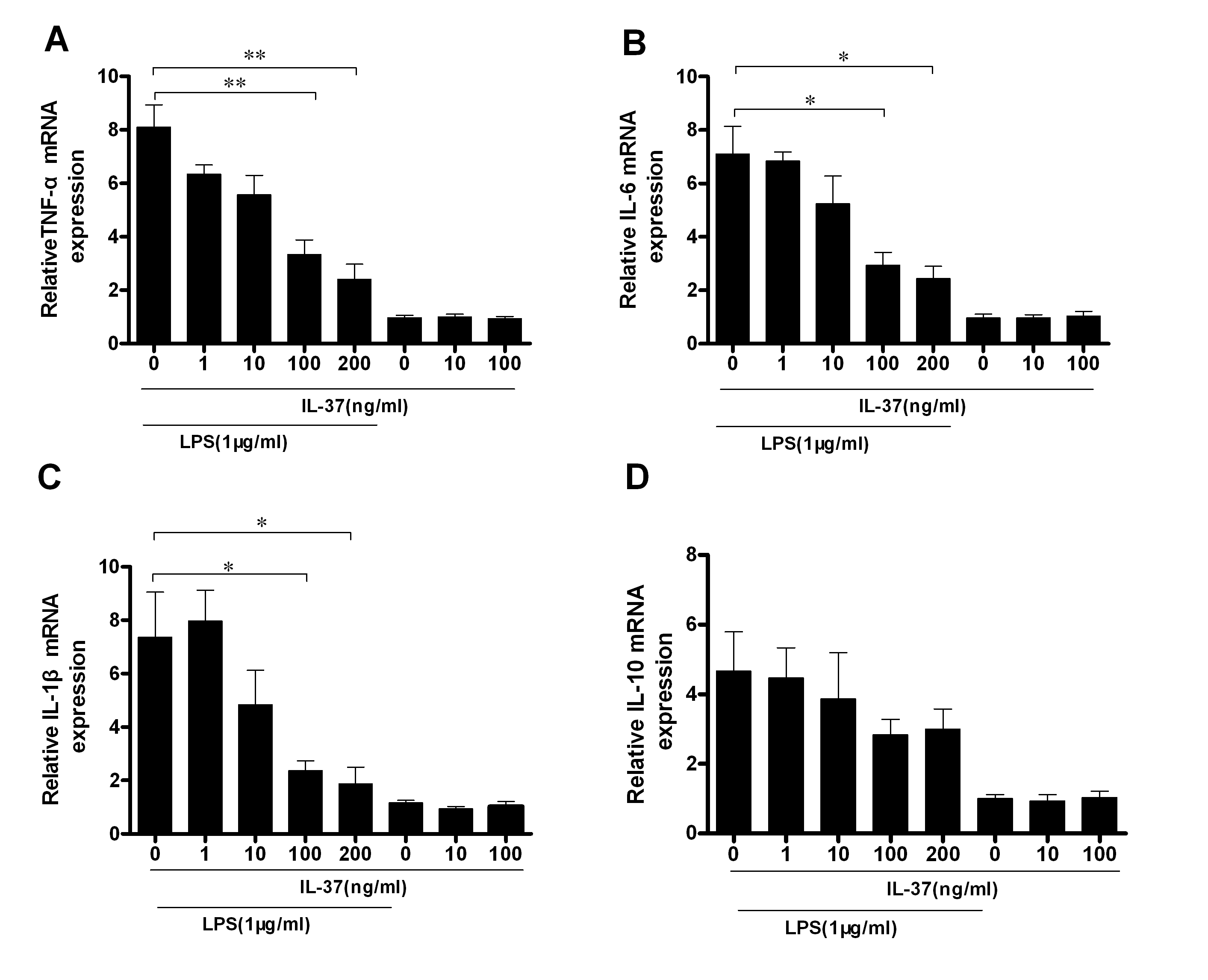

Supplement: Additional file 2 — Dose-dependent effects of IL-37 on inflammatory cytokines mRNA expression in PBMCs of healthy donors. PBMCs of healthy donors were stimulated for 6 h with different concentrations of IL-37, and then incubated further with or without LPS (1 μg/ml) for 6 h. The TNF-α (A), IL-6 (B), IL-1β (C) and IL-10 (D) mRNAs expression was analyzed by real-time polymerase chain reaction (PCR). Values are the mean ± SEM (n = 3). *P < 0.05; **P < 0.01. [file 1479-5876-12-69-S2.tiff]
